# Re-conceptualizing free will for the 21st century: acting independently with a limited role for consciousness

**DOI:** 10.3389/fpsyg.2013.00920

**Published:** 2013-12-09

**Authors:** Gregory B. Bonn

**Affiliations:** Jeffrey Cheah School of Medicine and Health Sciences, Monash UniversityBandar Sunway, Petaling Jaya, Malaysia

**Keywords:** free will, prospection, motor control, default network, volition, planned behavior, conscious experience

## Abstract

This paper examines the concept of free will, or independent action, in light of recent research in psychology and neuroscience. Reviewing findings in memory, prospection, and mental simulation, as well as the neurological mechanisms underlying behavioral control, planning, and integration, it is suggested in accord with previous arguments (e.g., Wegner, 2003; Harris, 2012) that a folk conception of free will as entirely conscious control over behavior should be rejected. However, it is argued that, when taken together, these findings can also support an alternative conception of free will. The constructive nature of memory and an integrative “default network” provide the means for novel and creative combinations of information, such as the imagining of counterfactual scenarios and alternative courses of action. Considering recent findings of extensive functional connections between these systems and those that subsume motor control and goal maintenance, it is argued that individuals have the capability of producing novel ideas and translating them into actionable goals. Although most of these processes take place beneath conscious awareness, it is argued that they are unique to the individual and thus, can be considered a form of independent control over behavior, or free will.

An identifying characteristic of human experience is a distinct, intuitive sense of volition. An inner voice existing within each of us insists that our actions result from personal will, telling us that we consciously choose our actions and rationally guide ourselves through life. Although intuitively satisfying, this notion has, deservedly, been the subject of much debate over the years. Recently, as neuroscience and psychology have expanded our understanding of the mind, a number of eminent scholars in these fields have provided evidence that contradicts such intuitive conceptions (e.g., Libet, 1985; Wegner, 2002; Wilson, 2002) leading some to argue that the notion of free will should be discarded entirely (e.g., Harris, 2012). Here I will argue that, although recent evidence does justify rejecting the idea that humans possess entirely conscious, reasoned self-control; *if one moves away from simplistic notions of consciousness and its role in human decision making*, *findings from several areas, when taken together, suggest an alternative conception of free will: Humans can possess levels of autonomy even if the processes involved are not entirely accessible to conscious reason.* This argument consists of several major components: In sections The Problem with Memory: Looking Backwards or Forwards? through Novel Conceptions I review evidence from multiple lines of research for novelty or creativity in human thought. Sections The Problem with Conscious Will and Why Consciousness? discuss findings regarding the limitations of conscious awareness as well as its potential value. Sections Internal Control of Behavior and Translating Simulations into Goals present evidence for the internal control of behavior as well as for functional connections between this control system and the systems involved with creative processing and counter-factual thinking. Finally, in sections Modeling Volitional Processes and Free Will with Limited Consciousness Awareness, I suggest a model for understanding how these systems interact and a more practically defensible model of free will: The creative integration of conceptual elements into novel, counter-factual simulations, and the use of these generative processes to guide behavior.

## Scope and limitations

The terms free will and consciousness both mean many things to many different people. For this reason it is important to first clarify some of the underlying assumptions that drive this argument. After establishing the gist of how these terms are used, as well as the intention of this paper, the line of thinking that follows should be clearer.

## What is meant by free will?

This paper is not meant as a philosophical treatise. As such, it does not address the more metaphysical issues, such as determinism, often invoked in free will debates. The point of this discussion is to present psychological evidence pointing to the possibility of individual humans behaving in novel and creative ways, albeit within the constraints of whatever context they live in. The human brain, it is presumed, can only process information that it is exposed to. The argument here is that human brain functions allow for given sets of information to be combined in novel or creative ways, and furthermore, the integrative conceptions created through these processes can be used to direct action.

Another point is that, although philosophers have significantly more nuanced ways of understanding the term, psychologists tend to operationalize free will by relating it to self-report (e.g., Libet et al., 1983; Wegner, 2008) which requires a form of self-reflective conscious awareness. The implicit condition is that one must be able to report upon all the processes leading up to a decision or behavior in order for it to be “free,” and conversely, if my brain generates an idea or initiates an action without my conscious awareness it is somehow not “me” doing the thinking or acting. The conception of freedom argued for here, on the other hand, merely requires that thoughts and resulting actions be novel and internally generated, that they result from a combination of experiences and characteristics which is unique to the individual. Unconscious, or implicit, processes are, in this view, essential components of how an individual processes information: Regardless of whether a particular process can be observed and narrated by the conscious, self-aware part of the brain, it can still make unique and important contributions toward thought and action, and thus, to the independence of the individual. *The arguments here, thus, specifically reject the simplistic notion that free will requires complete conscious awareness of the processes involved.*

## What is meant by consciousness?

A separate, but closely related point should also be made about the term “consciousness” which is used rather haphazardly by both scientists and laypersons to refer to a range of different phenomena (Chalmers, 1995). A basic conception of consciousness is experiential awareness or alertness, which is defined in contrast to unconsciousness (e.g., being awake as opposed to being in a deep sleep or a coma). Consciousness, from this viewpoint, can exist on many levels and possess varying degrees of complexity, all of which are simply characterized by the presence of subjective or phenomenal experience (Velmans, 2009). For example, every person experiences many degrees of alertness, a variety of emotions, as well as many other subjective phenomena over the course of a given day. There is no one conscious state that can be defined, there are rather, many overlapping, but differentiable states, some simple and some quite complex; some describable and some inscrutable. By this definition, although their subjective “experience” would vary tremendously (see, for example, Nagel, 1974) most forms of animal life as well as very small children would possess consciousness in some form. Tononi's (2008) conceptualization of consciousness as integrated information is suggestive in this regard. By contrast, studies that are commonly cited as evidence against conscious free will (e.g., Libet et al., 1983; Wegner, 2003) tend to operationalize consciousness as an awareness of mental processes which is measured through self-report: Participants are asked to describe aspects of their experience after performing certain tasks. It should be obvious that, although they are often conflated, these conceptions of consciousness are far from equivalent. The latter is more accurately a form of metacognition (i.e., “thinking about thought”), or self-consciousness, which is a small, so far as we know distinctively human, subset of the former (e.g., Rochat, 2003). Nevertheless, since in most discussions of free will, the term *consciousness* refers to this type of narrative self-awareness it is used here in the same way, although it is with the assumption that the individual experiences and interacts with his surroundings on multiple levels, only some of which are accessible to conscious self-report (e.g., Morin, 2006). For the purpose of this discussion, internal processes belong to the individual regardless of where they fall on any continuum of consciousness to unconsciousness.

## Memory, prospection, and creativity

Several areas of research have recently converged on a conception of memory as constructive, with the ability to combine elements of different remembered events in an integrative fashion. Evidence for processes that extract and recombine elements of multiple representations in generative, potentially novel, ways is discussed throughout the next six sections.

### The problem with memory: looking backwards or forwards?

Research has, over the years, pointed toward inaccuracies in the functioning of human memory (e.g., Schacter, 1999; Moscovitch et al., 2006; Addis et al., 2007). Remembering, it has been shown, is more of a creative, constructive process than the precise recall of past events. People, for example, often remember things that never happened (Roediger and McDermott, 1995), and conflate different episodes with each other, combining bits and pieces of various events into a single recalled instance (e.g., Tulving, 2002; Schacter and Addis, 2007a). Similarly, memories for specific events apparently change with each recollection, often incorporating current information with past impressions (Bridge and Paller, 2012). Having experienced problems stemming from such inaccuracies, it is not surprising that most people interpret such inaccuracies as “bugs,” or errors, in a memory system which should, intuitively, provide accurate information about the past.

Recent interpretations of such phenomena, however, (Dudai and Carruthers, 2005; Schacter and Addis, 2007b) speculate that the lack of factual accuracy in our recollections may instead be the signature of a system that evolved, not to store accurate representations of the past, but instead, to provide a means of flexibly imagining the future, as well as conceiving of other hypothetical scenarios. Surviving in the real world does not depend upon accurate recall of every past detail as much as an ability to predict future contingencies (Schacter et al., 2008). A system that can integrate details of multiple past events and is more sensitive to broad patterns and associations rather than accurately representing minutia would be well suited to this purpose.

The idea that memory systems play an important role in the creation of counter-factual scenarios, or formulating mental simulations, is supported by findings from several areas. Neurological studies find considerable overlap of brain systems used in memory and simulation (e.g., Buckner, 2010). Research on Construal Level Theory (Trope and Liberman, 2010) has identified similar patterns in how we conceptualize the past and the future. Also, studies of prospection, or predicting the future (Gilbert, 2006; Gilbert and Wilson, 2007), demonstrate that emotions and memory play an important role in imagining the future. These points are briefly reviewed in the following sections.

### Neurobiology of memory and prospection

Patients with memory deficits have, for some time, been observed to have difficulty planning for and imagining the future (Tulving et al., 1988; Hassabis et al., 2007). This led to some early speculation about a relationship between memory and prospection (e.g., Fuster, 1989) which has only recently been confirmed by functional imaging studies (Addis et al., 2009). Growing evidence points to a core network of brain regions involved in remembering the past and imagining the future, as well as other forms of mental *simulation* (Arzy et al., 2009; Spreng and Grady, 2010). In broad terms, tasks related to “mental time travel” (i.e., remembering the past and predicting the future) incorporate memory systems in the medial temporal lobes, the lateral parietal lobes and the hippocampal formation (Wheeler and Buckner, 2004), in addition to areas in the medial frontal lobes which are involved in perspective taking and theory of mind, or understanding others' mental states (Gallagher and Frith, 2003). It seems that many forms of self-projection; imagining the past and future, navigation (imagining the self in different physical locations) and theory of mind (taking the perspective of other people) depend on this same core network of memory-related brain areas (Buckner and Carroll, 2007).

### The default network: integrating information while at rest

Particularly relevant for this discussion are findings (e.g., Buckner et al., 2008) noting increased activity in this core network of brain regions during periods of undirected mental function, or passive states—hence its common designation as the “default network.” This default network is broadly associated with many forms of stimulus-independent thinking or internally focused cognition (Spreng and Grady, 2010): Mind-wandering, daydreaming, imagining the future, reminiscing about the past, as well as thinking about the cognitive states of others are all subsumed within its functions (e.g., Hassabis and Maguire, 2007; Schacter et al., 2008). When the brain is not occupied with processing external stimuli, activity reverts to this area where stored impressions are consolidated and reorganized (Buckner, 2010). The default network seems to facilitate the internal experience of scenarios and perspectives that transcend simple recall, and it seems to do so automatically through making connections between, or recombining, elements of multiple memory traces.

### Abstraction and the extraction of gist elements

Evidence of how this reworking of stored memories operates can be seen in Trope and Liberman's 2010) research on mental representations (summarized as Construal Level Theory). They have shown that mental representations of objects and experience become reconstructed in different ways at various levels of abstraction. “Psychological distance,” for example spatial, temporal, or social separation, between a person and an object leads to representations of differing resolution. Objects, people, and situations that are imagined to be distant are represented with less resolution; their conceptions become more abstract and essentialized (Liberman et al., 2002). A chair, for example, could be imagined as a specific object one is sitting on now, or imagining various future or past scenarios representations can take on numerous forms. For example, imagining a chair in an office, a living room, a restaurant, a car, or a spaceship can result in many different images. All that remains constant is the essential quality of affording sitting (see Gibson, 1977). The mind changes how it represents objects based upon the imagined context (e.g., Smith, 1998), adding or removing non-essential elements to facilitate the formation of a sensible scene. Complementing the research on memory and the default network, Construal Level Theory illustrates how gist elements from numerous impressions can be combined to create mental simulations placing the self and others in various situations and contexts (Wakslak et al., 2008).

### Emotions, memory, and imagining the future

Similarly, the integration and fuzzy processing that seems to occur in the default network is evident in Gilbert's (2006) writings about prospection. Emotional aspects of memories and current states influence mental simulations in important ways. Current emotional states, for example, color our emotional feelings about future events (Gilbert and Wilson, 2007); so, for example, if we are in a good mood when imagining a future date we are more likely to imagine it going well. Also, as memories tend to be skewed toward emotional peaks and valleys (Morewedge et al., 2005); simulations become “over emotionalized.” They tend to focus on brief highlights (or lowlights): Thinking about a future trip to an amusement park, for example, we might just remember the thrill of riding a roller coaster, and the pleasure of eating ice cream from past visits; not so much the monotony of waiting in long lines.

Gilbert (2006) also points out that memories and predictions of the future are strongly influenced by cultural scripts or “memes” (e.g., Blackmore, 2000; Bonn and Tafarodi, 2013). Our memories of the past, as well as imaginings of the future, are given meaning and form by the narratives that predominate in our cultures. The stories that we observe and hear being told from day-to-day shape our expectations and evaluations of our own lives, leading us to reshape the way we remember experiences over time (Klaaren et al., 1994). Memories (and in turn our expectations about the future) get rewritten each time they are accessed. Thus, over time the way we remember our past and what we expect in the future tends to fall in line with the narrative zeitgeist.

### Novel conceptions

Evidence, thus, supports the notion that as processing becomes removed from current surroundings representations change in nature: Details fall away while meaning, feelings, and connecting relationships become more important. Elements of different scenarios, when stripped of context, mingle with one another, potentially combining in ways many steps removed from actual experience.

Regardless of where such connections appear on a spectrum of conscious awareness, the integrative systems that seem to center around the default network allow for a great deal of flexibility in imagining and simulating possible realities. Although such simulations can be deeply flawed in the sense of being factually inaccurate and susceptible to bias, they are unique to the individual in that they are based upon that person's specific set of experiences. Each mind has a specific store of knowledge to which it can “add value” by integrating that information in qualitatively new ways (e.g., Tononi, 2008). New formulations of knowledge, in theory, can subsequently be fed back into the processing system and form the bases for new phantasies (e.g., Kashima et al., 2007). Simulations thus, have the potential to build upon each other through a process of scaffolding, feeding back into memory and integrating with each other iteratively over time.

Buckner (2010) actually takes the potential for originality one step further by arguing that, not only can we extract and combine elemental properties of information in creative ways, but random variations in neural firings would almost certainly play a part in the flexibility of this sort of system. He observes that seemingly random properties of neural systems are observable in nature. Aronov et al. (2008), for example, has identified a specific brain structure in finches that seems to relate to random song patterns. Cisek and Kalaska (2005) observed apparently random variations in neural firing influencing behavioral choice in monkeys. Relatedly, Bim (2012) has noted that physiological noise, such as respiratory and cardiac fluctuations, can influence resting functional connectivity in the brain. Thus, intrinsic properties of neural systems combined with environmental variation could allow for novel leaps in connectivity or new combinatorial patterns. Again, this is not an argument for conscious control over how the mind produces concepts, but for a capacity of originality, generativity, or creativity, in how it processes information.

## Consciousness: limitations and capabilities

### The problem with conscious will

The studies most commonly cited in neuropsychological arguments against the existence of free will began with a series of experiments conducted by Benjamin Libet and his colleagues (1983; 1985). Libet showed that readiness potentials or neural indications of an impulse to act were evident in participants' brains well before they reported any conscious intention to act. The conscious awareness of an intention to act in these experiments apparently came into being several 100 ms after the action had been set in motion within the brain. More recently, functional magnetic resonance imaging studies (e.g., Soon et al., 2008) have detected neural correlates of an intention to act many seconds before conscious awareness of that intention is reported. Conscious awareness of an impulse to act, thus, seems to be a neurological afterthought to the impulse itself.

Along similar lines, Wegner (2003) has collected extensive evidence indicating that, in many cases, our experience of conscious will is misleading. In some cases the feeling of causing or of willing an action does not exist after we have performed it (e.g., Geschwind et al., 1995) and in other cases we can be led to believe that we caused an action that we, in fact, did not (Ansfield and Wegner, 1996; Wegner and Wheatley, 1999). Wegner theorizes that the sense of having willed an action is inferred from various indicators: If we think a thought just prior to an action; the action is consistent with our thought; and there is no other obvious cause of the action; then, we tend to infer that we performed the action (Wegner, 2003). Even more, once we have inferred responsibility for an action we tend to rewrite our perceptions so that they are more consistent with this sense of authorship. Evidence shows, for example, that we estimate the gap between thought and action to be smaller for actions that we believe we have willed and longer for actions we do not feel responsible for (Ebert and Wegner, 2011). Again, the gist of these findings is that our feeling of having consciously willed an act is illusory in many ways. It seems that the conscious awareness of intention that we place so much weight upon, that we naively think of as causal, is, in fact, a narrative construction that is formed well after the train of causation has been set in motion.

What Wegner, Libet, and others have shown clearly is that the narrative awareness of a will to act arises after the actual impulse to perform a certain action. This does not mean, however, that the action is not owned by the person. It merely shows that action is not initiated by the narrative self. Only if our definition of the self is limited to narrative capability can we say that the person didn't initiate the action. One must acknowledge, based on the evidence, that the stories we create about our actions are misleading. They are subjective impressions, not factual accounts of all the processes involved. The argument can still be made, however, that the individual (i.e., the person in the broader, not necessarily self-conscious, sense) may initiate or control behavior on other, less explicit, levels.

### Why consciousness?

Libet (1999) himself pointed out that even if the conscious impression of will is merely corollary to and not the direct cause of an action it still occurs enough in advance of the action to allow for a conscious “veto” or a decision to not perform the action. Such late inhibitory decisions apparently involve an area in the frontomedian cortex (Brass and Haggard, 2007) and involve perceptual feedback (Moore et al., 2009). Consciousness, in this way, seems to have the potential to play some role in self-monitoring processes (Kuhn and Brass, 2009). Although we are not consciously aware of what is going on at every stage of the chain of neural events leading to action, there is room for a degree of conscious involvement if only to pull the emergency brake before it is too late. Thus, although it may not be the initial source of motivations and behavioral impulses, the part of the mind that is self-reflective; that can envision the self in causal and narrative contexts, may serve important monitoring and control functions.

Even Wegner, who has tirelessly argued that the folk understanding of conscious will is an illusion, has suggested that such an illusion probably serves some social purpose (Wegner, 2008). Being able to observe our behavior and its results in context, he suggests, allows individuals to better fit into complex social arrangements. Although illusory, the perspective of agency allows the brain to fine tune its behavioral impulses. “It tells us what we can and cannot do (Wegner, 2008; p. 241),” and further, the illusion of conscious will “makes behavior more open to modification (Wegner, 2008; p. 243).” So, for Wegner, the fact that *conscious* will is largely illusory does not completely rule out self-reflective capabilities from having some effect on behavior.

Consciousness may play an important role in monitoring the self and its behavior within contexts. Consciousness, in the sense of self-reflection, is closely entwined with the creation of narrative meaning (McAdams, 2008). Narrative meaning making involves processes such as conceptualizing the self in relation to higher-order or longer-term goals and social rules as well as imagining the consequences of actions and the reactions of others to those actions. This is all critical to understanding how the self relates to the surrounding world, and especially to integrating behavior with complex social contexts (Cozolino, 2002; Baumeister, 2008; Rochat, 2009). A monitoring function for the conscious self would, in theory, track behavioral impulses and their potential results, looking for conflicts between the actor and his longer term goals as they arise, with the potential for triggering inhibitory functions at various times. Consciousness, in this view, influences behavior by providing broad contextual input and inhibitory feedback into a complex planning system. Behavioral impulses, however, would be produced by mechanisms that are outside of *direct* conscious control. Consciousness *per se* is most useful because it can monitor behaviors, goals, and the changing environment in real-time watching for potential conflicts. The bulk of processing, however, must take place in other complex systems that operate largely beneath the surface. Consciousness seems most important for providing up-to-the-minute contextual integration and feedback to other systems. Self-reflective monitoring facilitates the fine-tuning of impulses and behaviors, and inhibitory control, necessary for high-level integration with dynamic physical and social environments.

## Control and planning of behavior

Strictly conscious control over behavior seems to be ruled out by our improved understanding of the mind. Does this mean, however, that a person is not in control of their behavior? Again, keeping in mind a broad definition of the “person” as including both conscious and unconscious elements, recent discoveries can shed light on this issue. First, there is a separate motor control network dedicated to internally generated, voluntary, goal oriented behaviors as contrasted with externally-triggered and more habitual behaviors. Second, there appear to be connections between the default network, where novel ideas and counterfactual scenarios are produced, and this goal-oriented control network that allows for the internal generation of action.

### Internal control of behavior

Two major sources provide activating input to the primary motor cortex, which is the initiator of muscle movement (Haggard, 2008). The first motor control system runs from the sensory cortices to the primary motor region via the pre-motor area: Activity in these areas relates to stimulus-driven, or reflexive, responses to sensory input as well as to habitual behaviors such as grasping, eating, and walking which are performed largely unconsciously (Prabhu et al., 2007). The second motor system involves multiple regions, including the cingulate, frontal cortices, and basal ganglia, which connect to the primary motor cortex via the pre-supplementary and supplementary motor areas. Behaviors that require planning and goal maintenance engage some or all of this system (Daw et al., 2006; Hirosaka, 2008). Processes mediated by pre-supplementary motor area (preSMA) connections generally allow for the flexible, online integration of goal states, decisions, and action priorities with feedback from the environment. Imaging studies, for example, show that the preSMA is consistently involved in task-focused activities and situations that require the preferential selection of certain behaviors over others (Nachev et al., 2007). Importantly, patients with damage to the preSMA are deficient in their ability to prioritize behaviors and suppress automatic behaviors (Pacherie, 2007): They might, for example, impulsively grasp, eat, or drink without reporting the intention or desire to do so (Della Salla et al., 1991), suggesting that the preSMA plays a role in inhibiting the habitual behaviors governed by the first motor control system.

This second motor control system plays a crucial role in tasks related to goals and decision making. The preSMA, along with the frontopolar cortex and the rostral cingulate, is active in tasks requiring decisions between multiple options, such as choosing between right or left hand key presses (Ammon and Gandevia, 2007; Mueller et al., 2007). The frontopolar cortex is also involved in maintaining goal states such as suppressing responses to immediate environmental demands (Koechlin and Hyafil, 2007; Dreher et al., 2008) and, along with the anterior cingulate (ACC), is seemingly involved in the production of goal-directed action sequences (Holroyd and Yeung, 2012). The ACC, through the preSMA, also seems capable of selecting and initiating action in the absence of external prompts, as well as monitoring and adjusting those actions in response to feedback (Rowe et al., 2010; Zhang et al., 2012). All told, there are extensive findings indicating that the preSMA is involved in interfacing multiple goal and decision-related subsystems with the primary motor cortex.

Most complex behaviors would involve an integration of these two motor systems, with the more automatic system triggering the basic movements and the decision and goal related system throwing in guidance and inhibitory impulses at important junctures. Although these relationships need further clarification, the pathways mediated by the supplementary and pre-supplementary motor areas do seem to allow for basic internally guided choice, selective inhibition, and “volition-like” control of behavior (Haggard, 2008), though probably not complex decision-making or reasoning (e.g., Koechlin and Hyafil, 2007). The next section will propose that these systems are capable of interfacing with the default network during planning tasks. In this way, output from integrative processes taking place in the default network could be incorporated into goal formation and behavioral control.

### Translating simulations into goals

To this point we have established two important concepts. First, processing in the default network allows humans to create novel combinations of information. Information stored in memory is broken down to elemental form and connections made between elements during times of reduced sensory input. This allows for patterns and relationships among multiple impressions to be extracted and for the flexible generation of counterfactual simulations. Second, faculties exist for internally maintained goals to exert flexible control over behavior. Humans can replace automatic, reflexive behaviors with internally guided, goal-directed action.

Default network functioning, by definition, is most active when the external attention necessary for goal-oriented functioning is absent. Thus, the systems that produce novel ideas and those that maintain goals are usually thought of as contradictory, or negatively correlated (e.g., Fox et al., 2005; Carhart-Harris and Friston, 2010). Free will as it is conceptualized here, however, would require an interface between these two levels of operations. For behaviors to be called free, or independently generated, they would need to result not just from the complex training processes that reside within the goal maintenance system: They would need to incorporate elements that are unique to the individual; that are novel and creative, as well. The brain would need to be able to translate the abstract simulations and integrated information produced by the default network into actionable goals.

Recently, Spreng et al. (2010) found that tasks in which participants made goal-related plans activated default network regions as well as regions commonly associated with cognitive control (i.e., areas of the frontal cortex and the ACC). Similarly, other recent studies (Gerlach et al., 2011; Spreng and Schacter, 2012) have found that solving imagined future problems involved default network areas as well as control network areas. In particular, the dorsolateral prefrontal cortex, which is central to rule acquisition and goal maintenance functions (Badre and D'Esposito, 2009; Badre et al., 2009; Packer and Cunningham, 2009) has been implicated, along with default network regions, in planning tasks. Gerlach et al. (2011) have also found functional connections between the posterior cingulate cortex, which is thought to be the nexus of the default network, and the dorsolateral prefrontal cortex. The default network, then, is seemingly able to interface with goal maintenance and cognitive control functions when engaged in problem solving and future planning. This suggests that the generative capacity of the default network; the ability to extract elements of diverse memories and impressions and integrate them in novel ways, could be used to create concrete, actionable goals.

## Reconceptualizing free will

### Modeling volitional processes

**Figure 1:**
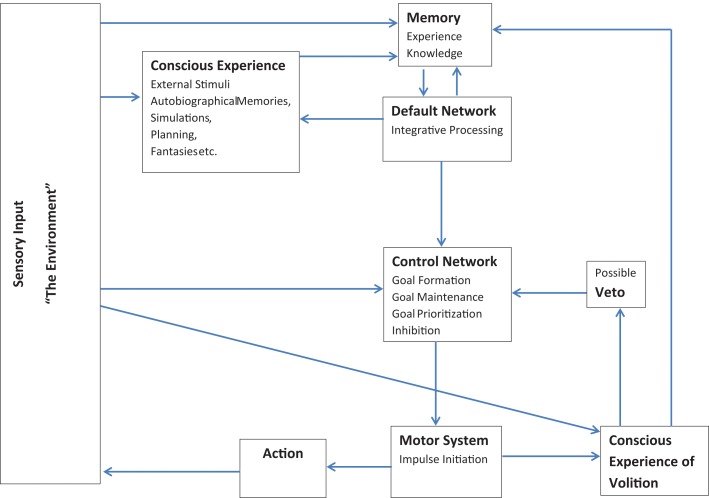
**Model showing interconnections among systems for memory, mental simulations, behavioral control, and conscious awareness**.

Thus, although the folk conception of free will as entirely conscious self-control seems to be dead in the water (e.g., Wegner, 2008; Harris, 2012), I have argued here for a broader conception of free will which is compatible with current neuroscientific understanding. This model is broadly outlined in Figure 1: Essentially, according to this model, the individual can generate novel concepts, translate those concepts into goals, and initiate and monitor activity toward achieving those goals. The model consists of two feedback loops which are largely anticorrelated. The first loop is made up of the default network and memory systems: The default network extracts elements of stored information from memory systems, integrates and combines information in various ways, and feeds the results back into memory. The second loop involves the executive control and motor networks: Executive regions establish and update goal priorities, while initiating and monitoring motor activities according to environmental feedback. These two loops are linked via a feed forward connection from the default network to the executive control network (e.g., Gerlach et al., 2011) which allows for output from simulations occurring within the default network to be uploaded into the executive control network and incorporated into the creation and maintenance of externally directed goals. Additional information is continually fed back into the memory and control systems through sensory input from the environment. Each person's activities lead through their observed effect on the environment to a unique store of information in the individual memory system. The many impressions; sensory, emotional, or otherwise, that the individual has in memory are available to the default network during times of reduced input. During such periods, elements or traces of different memories are combined together in various ways with the results then stored and available for further processing.

Both loops are accessible to a limited degree by conscious awareness or self-reflective abilities and receive some feedback from this conscious level. Conscious awareness is mostly important here for online monitoring and the incorporation of current contextual information. Although most processing occurs beneath conscious awareness, the ability to direct attention both inwards and outwards could make conscious awareness especially useful for monitoring and minimizing conflicts between actions, intentions, and real-time, current context. In the case of the control/motor network loop, conscious awareness also possesses some veto-like inhibitory powers: It can interfere with certain impulses before action is initiated (e.g., Libet, 1999). In the context of the default network, conscious monitoring could integrate current contextual information with ongoing simulations, perhaps providing reality checking functions which keep simulations more in line with the external environment. In both systems conscious awareness need not be directly causal, but just provide monitoring and additional real time information as feedback into the system.

Though preliminary, this model provides an outline of how a practical form of free will, independently generated and controlled activity, can exist and be consistent with current findings from psychology and neuroscience. Those who adhere to particular definitions of “free will” could certainly take issue with these arguments. However, if one accepts that free will can exist in degrees limited by a person's knowledge and experiences; and, that decisions do not need to be *entirely* conscious in order to be owned by the individual. Then, I believe there is evidence to posit a level of will and independence within the person. Individuals can integrate information creatively to conceptualize multiple different scenarios or goals; they can choose between options; they can act according to goals; they can abort actions if they do not match current goal sets; and they can incorporate and integrate information from ongoing feedback into subsequent simulations and decisions.

### Free will with limited consciousness awareness

As I have discussed, the role that consciousness, as it is commonly conceptualized, plays in these processes is limited. It is not, however, non-existent. There is a place in this model for conscious monitoring of simulations and goal states, the integration of sensory information with ongoing internal processes, and related inhibitory control. The vast majority of processing in this model does, however, take place beneath the level of conscious awareness and self-report. If one considers the degree to which unconscious processes are involved in every action that we undertake, this should become far less of a concern. For example, consider everyday acts like walking from one place to another, or speaking a sentence. These are incredibly complex behaviors requiring the coordinated operations of many thousands of neurons and muscles simultaneously. When we perform such acts we are not aware of exactly how we balance our bodies or shape our mouth and tongue at any particular moment. Our bodies just perform as we expect them to (normally) and we report via our conscious awareness a summary of what we did or what we intended to do: We just think “I walked to the café and ordered a coffee,” for example. We don't notice exactly how every muscle moved along the way, where we placed our feet, or how we formed our words. Most people, however, would not claim a lack of control over their bodies. It is not irrational to believe that, yes; *I* took a walk and ordered coffee.

Cognitive psychology has shown over the years that large portions of mental processing take place beneath the level of consciousness awareness (e.g., Kahneman, 2011; Kihlstrom, 2013). Much of our mental processing involves energy- and time-saving shortcuts, and much of our behavior is, for similar reasons, habitual. This does not necessarily mean, however, that consciousness is entirely left out of the picture, but certainly the conception that we are in complete conscious control (or that we always behave rationally) has been proven to be illusory. What I have argued here is that if we abandon the ideas that human will needs to be completely explicit, and that conscious awareness and control of every process is required for an individual to be a decision maker, it is possible to see evidence of originality, individuality, and creative processes, as well as cognitive control, in the way that each person thinks and behaves. This type of individuality, I believe, can be called free will.

### Conflict of interest statement

The author declares that the research was conducted in the absence of any commercial or financial relationships that could be construed as a potential conflict of interest.
